# Prevalence of dyslipidemia and the association with levels of TSH and T4 hormones among patients in south region of Jordan

**DOI:** 10.5937/jomb0-40504

**Published:** 2023-10-27

**Authors:** Omar Mohammad Atrooz, Mazen Nayef Hiresh, Alghonmeen Reham Dlewan, Mohammad Omar Atrooz, Ghofran Nayef Hiresh, Aseel Mahmoud Alasoufi, Ihssan Omar Atrooz

**Affiliations:** 1 Mutah University, Department of Biological Sciences, Mutah, Jordan; 2 The Shrewsbury and Telford NHS Trust, Princess Royal Hospital, Emergency Department, Telford, United Kingdom; 3 Resident in Jordanian Royal Medical Service, Anesthesia and Intensive Care Unit, Amman, Jordan; 4 Al-Tafilah Health Directorate, Al-Tafilah, Jordan; 5 Ma'an Governmental Hospital Laboratory Development, Ma'an, Jordan; 6 Al-Karak Governmental Hospital, Al-Karak, Jordan

**Keywords:** hypercholesterolemia, low HDL, hypertriglyceridemia, high LDC, fasting blood glucose, TSH, T4, hiperholesterolemija, nizak HDL, hipertrigliceridemija, visok LDC, glukoza u krvi natašte, TSH, T4

## Abstract

**Background:**

Glycolipid metabolism disorders (dysglycolipidemia) are characterized by elevated levels of glycolipid profile components and fasting blood glucose. Dysglycolipidemia are major threats to human health and life. Therefore, the aim of this cross-sectional study is to estimate the prevalence of dysglycolipidemia and the existence of association of TSH and T4 and glycolipid profiles.

**Methods:**

Cross-sectional data were obtained from the medical laboratory of Ma'an Governmental Hospital. A total of 141 patients' results were collected (18-60 years). Differences in the glycolipidemic profiles according to age and sex and TSH and T4 were compared. Different statistical analyses were used to analyze the prevalence of dysglycolipidemia and the correlation with the levels of TSH and T4.

**Results:**

The study involved results of 141 patients (54.7% males and 45.3% females) in Ma'an Province (Jordan), who visited the internal medicine clinic at Ma'an Governmental Hospital. Patients have overweight and BMI of more than 25 kg/m2. The overall results of the prevalence of dyslipidemia indicated that patients have 42.5% of hypercholesterolemia, 48.2% of high LDL-C, 34.1% of hypertriglyceridemia, and 41.8% of low HDL-C. The prevalence of isolated lipid profiles showed that 10 patients have mixed dyslipidemia. The association of dyslipidemia with age indicated a positive significance between triglyceride and older people (≥40 years), while HDL levels have a significance with gender (p=0.025). The overall ANOVA model yielded non-statistical significant results between levels of any components of lipid profile and levels of TSH and T4 hormones. Welch test (p=0.036) showed positive significance between levels of fasting blood glucose and triglyceride levels.

**Conclusions:**

Our results showed and confirmed the presence of a high percentage of hyperlipidemia in Ma'an province and there was no relationship with levels of TSH and T4. A relationship exists between levels of triglycerides and blood glucose concentrations.

## Abstract (sr)

**Uvod:**

Poremećaji metabolizma glikolipida (disglikolipidemija) karakterišu povišeni nivoi komponenti glikolipidnog profila i glukoze u krvi natašte. Disglikolipidemija je glavna pretnja ljudskom zdravlju i životu. Stoga je cilj ove studije preseka da se proceni prevalencija disglikolipidemije i postojanje povezanosti TSH i T4 i glikolipidnih profila.

**Metode:**

Podaci o poprečnom preseku su dobijeni iz medicinske laboratorije Vladine bolnice Ma'an. Prikupljeno je ukupno 141 rezultata pacijenata (18-60 godina). Upoređene su razlike u profilima glikolipidemije prema starosti i polu i TSH i T4. Različitim statističkim analizama analizirana je prevalencija disglikolipidemije i korelacija sa nivoima TSH i T4.

**Rezultati:**

Studija je obuhvatila rezultate 141 pacijenta (54,7% muškaraca i 45,3% žena) u provinciji Ma'an (Jordan), koji su posetili kliniku interne medicine u Vladinoj bolnici Ma'an. Pacijenti imaju prekomernu težinu i BMI veći od 25 kg/m2. Ukupni rezultati prevalencije dislipidemije su pokazali da pacijenti imaju 42,5% hiperholesterolemije, 48,2% visokog LDL-C, 34,1% hipertrigliceridemije i 41,8% niskog HDL-C. Prevalencija izolovanih lipidnih profila pokazala je da 10 pacijenata ima mešovitu dislipidemiju. Povezanost dislipidemije sa uzrastom ukazala je na pozitivan značaj između triglicerida i starijih osoba (≥40 godina), dok nivoi HDL-a imaju značaj za pol (p=0,025). Ukupni ANOVA model je dao nestatistički značajne rezultate između nivoa bilo koje komponente lipidnog profila i nivoa TSH i T4 hormona. Velch test (p=0,036) pokazao je pozitivan značaj između nivoa glukoze u krvi natašte i nivoa triglicerida.

**Zaključak:**

Naši rezultati su pokazali i potvrdili prisustvo visokog procenta hiperlipidemije u provinciji Ma'an i nije bilo veze sa nivoima TSH i T4. Postoji veza između nivoa triglicerida i koncentracije glukoze u krvi.

## Introduction

Dyslipidemia is a condition characterized by elevated levels of cholesterol (Cho), triglycerides (TG), and low-density lipoprotein cholesterol (LDL), and maybe lower plasma levels of high-density lipoprotein cholesterol (HDL) [1]. Whereas a dysglycolipidemia is characterized by combination of dyslipidemia and elevated levels of fasting blood sugar (FBS) and hemoglobin A1C (HbA1c). In this condition, the increase may occur singly or in combination, and it is accompanied or associated with other serious diseases like cardiovascular disease (CVD), Diabetic Mellitus (DM), obesity, and others [2]. World Health Organization (WHO) indicated that the number of deaths and abnormalities related to dyslipidemia, CVD, DM or obesity increased annually [3]. Also, it suggested that treatment of these diseases results in a reduction in the number of deaths [4]. Dysglycolipidemia is a world population problem, and is prevalent in Arab countries especially Middle East populations [5].

Thyroid-stimulating hormone (TSH) and thyroxine hormone (T4) levels are primarily involved in energy homeostasis, hypertension, glucose and lipid metabolism. Abnormalities in thyroid hormone levels are associated with many diseases like CVD, DM, hyperlipidemia, metabolic syndrome, and obesity [6]. Thyroid and glycolipidemia dysfunctions are the two most common disorders with substantial overlap. Both are related to higher morbidity and mortality and thus impacts substantially on health care, worldwide [7]. Thyroid hormones play a vital role in glucose and lipid metabolism, blood pressure regulation, and energy consumption [8]. Previous studies found a relationship between abnormalities of levels of TSH and T4 and glycolipidemia [9], while other reports did not show significant associations [10] [11].

Many previous reports and studies have indicated a possible association between hypothyroidism and overweight [12], abnormal lipid profile [13] [14], atherosclerosis, endothelial dysfunction and diabetic patients [15]. Many studies review the effects of thyroid hormones on metabolism, as well as on various biochemical parameters [16]. Lambadiari et al. [17] reported the effects of thyroid hormones on glucose hemostasis, fasting glucose levels, and on impaired insulin secretion. Also, Dimitriadis et al. [18] and Klein et al. [19] reviewed the impacts on the cardiovascular system. Other studies showed the effects on blood pressure and Cho levels [20].

This study aimed to estimate the prevalence of dyslipidemia and DM and if there is any relationship between these parameters and levels of TSH and T4 hormones in patients living in Ma'an province, in the south of Jordan, in addition, to investigate the association between glycolipids profile and the patients gender and age.

## Materials and methods

### Study design

This cross-sectional study was conducted in Ma'an Province (south of Jordan) from January 2022 to May 2022.

### Setting and data collection

All data results of patients were collected from the medical laboratory at Ma'an Governmental Hospital with the ethical standards and agreement.

### Study size and participants

Medicals results from a total of 141 patients (male and female) who visited the internal medicine clinic, were collected from medical laboratory records according to the Hakeem program approved by the hospital and the Jordanian Ministry of Health. These patients have overweight and BMI more than 25 kg/m^2^, and aged from 18-60 years.

### Variables and measurement

Analysis of FBS, HbA1c, T4, TSH, TG, Cho, HDL-C, and LDL-C was done by Automated Clinical Chemistry Analyzer, Cobas C311, Serial number 16F2_20, Roche, Germany. Hormones (TSH and T4) determination were done by Cobas e411, serial number 6230_10, Roche, Germany, and hematological parameters were analyzed by Fully Automatic Celltac Alpha MEK-6510K Hematology Analyzer, Serial number 02997, Japan.

### Ethical issues

There were no ethical issues related to this study. The study was approved by the Ma'an Governmental Hospital Ethics Committee with the ethical standards and agreement, which was approved by the Council for Health Systems Accreditation and applied to the diagnostic services standards of the Council for Health Systems Accreditation in the Ma'an Governmental Hospital.

### Statistical analysis

The categorical data expressed in frequency and percentage, the scale data expressed in mean and standard deviation, Chi-square of independence test was used to explore the association between dyslipidemia with patient's age and gender, additionally multiple linear regression was used to investigate the impact glycolipids profile on patients' TSH and T4, moreover, MANOVA test was used to study mean differences of lipid profile according to gender, alpha level set at <0.05 considers statistically significant. SPSS IBM software ver28 was used to analyze data.

## Results

### Descriptive data

A total of 141 adult patients (Table 1) from Ma'an province participated in the study, more than half of the patients were females 80 (56.7%) compare to 61 (43.3%) were males, with mean study participants' age of 38.18±5.79 years. Regarding participants' clinical laboratory characteristics, the mean of TSH and T4 were found to be 3.52±1.11 mIU/L and 16.09±3.75 pmol/L respectively, Moreover, the participants' mean glycolipids profiles were 5.03±1.21 mmol/L for Cho, 1.6±0.90 mmol/L for TG, 1.30±0.34 mmol/L for HDL and 3.33±1.11 mmol/L for LDL, 5.68±1.08% for HbA1c and 6.33±2.05 mmol/L for FBS. On other hand, the complete blood count was investigated as well, the participants' mean Hb 150.13±28.7g/L, PLT 292.86±8.73 x 10^9^/L, MCV 82.02±6.22 fL, MCHC 330.13±15.3 g/L, RBC 4.92±0.58 x 10^12^/L and WBC 7.60±2.44 x 10^9^/L .

**Table 1:** Summary of participants’ characteristics.

Variables	Frequency	Percentage	Mean ± SD
Gender			
Male	80	56.7	
Female	61	43.3	
Age in years			38.18±5.79
Less than 40	77	54.6	
40 and above	64	45.4	
TSH (mIU/L)			3.52±1.11
T4 (pmol/L)			16.09±3.75
Cho (mmol/L)			5.03±1.21
TG (mmol/L)			1.6+0.90
HDL (mmol/L)			1.30+0.34
LDL (mmol/L)			3.33+1.11
HbA1c (%)			5.68+1.08
FBS (mmol/L)			6.33+2.05
Hb (g/L)			150.13+28.7
PLT (10^9^/L)			292.86+8.73
MCV (FL)			82.02+6.22
MCHC (g/L)			330.13+15.3
RBC (10^12^/L)			4.92+0.58
WBC (10^9^/L)			7.60+2.44

### Prevalence of dyslipidemia among study patients

The results in the Table 2 show that 34 (24.1%) and 26 (18.4%) of patients have a borderline and high Cho level, similarly 26 (18.8%) and 42 (29.8%) of them have a borderline and high LDL level, in the same context 17 (12.1%) and 31 (22.0%) of patients have borderline and high TG level, regarding HDL less than half of sample 59 (41.8%) have high risk compared to 82 (58.2%) having no risk.

**Table 2:** Prevalence of dyslipidemia among study patients.

Dyslipidemia	Category	Frequency	Percentage
Cho	Normal	81	57.4
	Borderline	34	24.1
	High	26	18.4
LDL	Normal	73	51.8
	Borderline	26	18.4
	High	42	29.8
TG	Normal	93	66.0
	Borderline	17	12.1
	High	31	22.0
HDL	No risk	82	58.2
	High risk	59	41.8

### Association of dyslipidemia with patients' age and gender

The chi-square of independence was used to explore if there is a significant association between patients' dyslipidemia according to gender and age, the results in the Table 3 revealed that there is an association between gender and HDL level, indicating that the females participants are significantly have a higher proportion of HDL level than male X^2^(1)=5.055, *p* *=0.025*, additionally a significant association between TG and age was noted indicating that the older people (≥40 years) significantly have a higher proportion of high TG level than <40 years old age people X^2^(2)=11.127, *p=0.004*. On other hand, neither Cho, LDL nor TG levels were significantly associated with patients' gender and neither Cho, LDL nor HDL level were significantly associated with patients' age (p>0.05 for all).

**Table 3:** Association between dyslipidemia with age and gender.

Dyslipidemia		Gender			Age/years		
		Male<br>n (%)	Female<br>n (%)	p-value	Less than<br>40 n (%)	≥40 n (%)	p-value
Cho	Normal	32(52.5)	49(61.3)	0.113	44(57.1)	37(57.1)	0.483
	Borderline	13(21.3)	21(26.3)		21(27.3)	13(20.3)	
	High	16(26.2)	10(12.5)		12(15.6)	14(21.9)	
LDL	Normal	28(45.9)	45(56.3)	0.374	41(53.2)	32(50.0)	0.922
	Borderline	14(23.0)	12(15.0)		14(18.2)	12(18.8)	
	High	19(31.1)	23(28.7)		22(28.6)	20(31.3)	
TG	Normal	42(68.9)	51(63.7)	0.314	59(76.6)	34(53.1)	0.004
	Borderline	9(14.8)	8(10.0)		9(11.7)	8(12.5)	
	High	10(16.4)	21(26.3)		9(11.7)	22(34.4)	
HDL	No risk	42(68.9)	40(50.0)	0.025	49(63.6)	33(51.6)	0.148
	High risk	19(31.1)	40(50.0)		28(36.4)	31(48.4)	

### Impact of glycolipids profile on patients TSH and T4

Six glycolipids lab tests namely (Cho, TG, HDL, LDL, HbA1c, and FBS) were entered into a multiple linear regression model, the aforementioned variables have explained only 3.2% of the variation in TSH, the overall ANOVA model yielded a non-statistical significant result *F* (6,140)=0.425, *p=0.862* indicating no one of the aforementioned variables has a significant impact on patients' TSH level (Table 4). In the same context, six glycolipids lab tests were investigated for their impact on patients' T4 as well and the aforementioned variables have explained only 5.3% of the variation in T4, moreover, overall ANOVA model yielded a non-statistical significant result *F* (6,140)=1.242, *p=0.289* indicating no one of aforementioned variables have a significant impact on patients' T4 levels Table 5.

**Table 4:** Impact of glycolipids profile on patients’ TSH.

Predictors	B coefficients	Std. Error	t-value	p-value
Cho	0.535	1.950	0.274	0.784
TG	1.357	1.114	1.218	0.225
HDL	2.978	3.087	0.965	0.336
LDL	0.379	1.825	0.207	0.836
HbA1c	0.493	1.033	0.477	0.634
FBS	0.239	0.365	0.655	0.514
F (6,140) =0.425, R2=0.032, p=0.862				

**Table 5:** Impact of glycolipids profile on patients’ T4.

Predictors	B coefficients	Std. Error	t-value	p-value
Cho	0.947	0.974	0.972	0.333
TG	0.800	0.556	1.438	0.153
HDL	2.480	1.541	1.609	0.110
LDL	1.192	0.911	1.308	0.193
HbA1c	0.367	0.516	0.712	0.478
FBS	0.065	0.182	0.358	0.721
F (6,140) =1.242, R2=0.053, p=0.289				

### Relationship between lipid profile components

**Figure 1:**
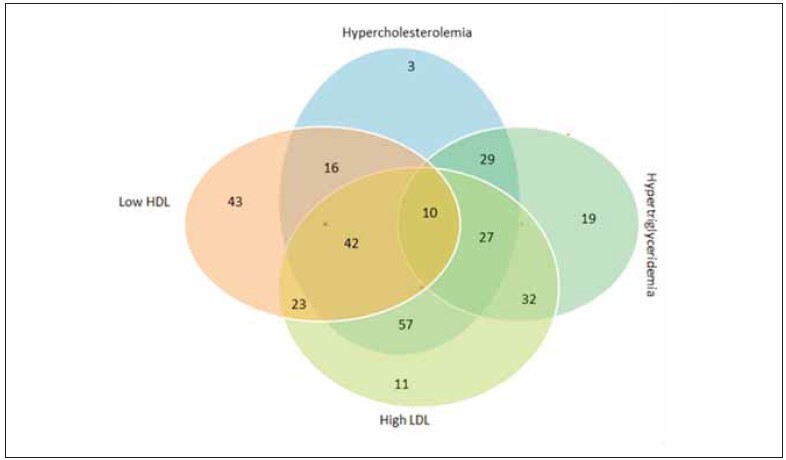
Venn diagram of the interaction between dyslipidemia components.

The Venn diagram illustrates the logical overlapped relationship between different types of dyslipidemia given that the patients borderline and undesirable levels were grouped. Figure 1 shows that 10 patients had hypercholesterolemia, hypertriglyceridemia, high LDL and low HDL, moreover, 42 patients accounted for low HDL, high LDL, and hypercholesterolemia, in the same context, 27 patients had hypercholesterolemia, hypertriglyceridemia, and high LDL.

### Correlation between lipid profile components and blood glucose level

The result in the (Table 6) shows that the level of FBS was significantly different according to TG levels (Welch test=3.607, p=0.036), and the Games-Howell post hoc test) yielded that those having high TG levels are significantly having a higher mean of FBS than those having normal TG levels p=0.045, while no statistically significant blood sugar mean differences were observed between other lipidomic profiles. In addition, neither Cho, LDL nor LDL levels were different according to FBS levels (p>0.05) for all.

**Table 6:** Association between lipidomic profile components and FBS levels.

Variables	Category	N	Mean	SD	Test value	p-Value
TG level	Normal	93	5.99	2.39	3.607^a^	0.036
	Borderline	17	5.72	0.91		
	High	31	7.69	3.80		
Cho level	Normal	81	5.94	1.66	2.557^b^	0.081
	Borderline	34	6.41	3.00		
	High	26	7.47	5.44		
LDL	Normal	73	5.92	1.66	1.806^b^	0.168
	Borderline	26	6.37	3.22		
	High	42	7.04	4.45		
HDL	High risk	59	6.16	2.45	0.561^c^	0.576
	No risk	82	6.46	3.43		
a= welch test, b = one-way ANOVA, c = independent T-test. All variables level units are in mmol/L.						

## Discussion

One of the problems of world public health is the increasing prevalence of lipid metabolic problems and DM. The prevalence differs from country to country according to socioeconomic, ethnicity, culture, and race [1]. This study on Ma'an province is considered the first that focused on the prevalence of dysglycolipidemia among patients living in this region.

The prevalence of dyslipidemia results showed that 42.5%, 48.2%, 34.1%, and 41.8% of borderline and high frequencies of hypercholesterolemia, high LDL, hypertriglyceridemia, and low HDL, respectively. Results of the association between dyslipidemia with gender and HDL level showed a significantly higher proportion in females than males (p=0,025), while others showed no significance and this finding is consistent with other studies [21]. According to age, the finding showed only a significant association between TG and age for patients more than 40 years old.

In our study, the prevalence of hyperlipidemia was about 34%. However, our findings on the presence of prevalence of dyslipidemia are consistent with those of previous studies conducted in China, KSA, Oman, Kuwait, Yemen, and Iraq [22] [23]. The percentage of the prevalence is higher than in Egypt (27.1%) and lower than in KSA (46.3%), and Iraq (38.5%) [24] [25]. On other hand, the prevalence of isolated biochemical parameters of lipid profile (as shown in the Venn diagram) showed an interesting overlapping relationship with 10 patients for all lipid parameters, and 42 patients have high Cho levels, low HDL, and high LDL. Similar increasing trends of dysglycolipidemia have been observed in many countries [26]. According to the National Cholesterol Education Program's Adult Panel criteria, and World Health Organization Asia-Pacific guidelines, the prevalence of diseases related to the abnormality of glycolipid metabolism is increasing [6].

One of our study purposes was to identify whether any association exists between TSH and T4 levels with one or more of the lipid profile components. Many previous studies reported that thyroid dysfunction affects glucose levels and lipid metabolism. Our findings indicated that these variables of glycolipid profile contents have only 5.3% variation in T4, and only 3.2% in TSH, Although, the ANOVA model indicates no significant results p=0.289 (for T4) and p= 0.862 for TSH. Association between lipidomic profile components indicated that statistically, only TG levels have a positive significant relationship with FBS levels. It was found that patients having higher levels of TG, also have higher levels of FBS. Our finding was consistent with other previous studies conducted in Korea, India, Saudi Arabia, and Ethiopia [24] [27]. The similarities might be that high concentration of TG may potentially contribute to insulin resistance, reduce the uptake of glucose, and may be due to physical inactivity [28]. Similarly, previous studies performed in Bosnia, Herzegovina [29], China, and Nepal [30], showed a positive correlation between TG levels and glucose concentrations. They related this correlation to insulin resistance, which decreases glycogen synthesis and protein catabolism while inhibiting lipoprotein lipase in adipocytes, resulting also in defects in fatty acid metabolism and increasing very low density lipoprotein (VLDL) [31].

In general, the high percentage of the prevalence of glycolipidemia in this region may be due to genetic factors and environmental conditions as this region is classified as a desert area. However, other factors like patients' living style, physical inactivity, smoking, and nutrition.

## Conclusion

This cross-sectional study is the first to be conducted and to illustrate the existence of dysglycolipidemia in patients living in the Ma'an governorate in the south of Jordan. The study focused on the presence of dysglycolipidemia and if there is any relationship with the levels of T4 and TSH hormones. The results showed that 34.2% of patients have dysglycolipidemia, and the percentage is higher in females than in males. Also, it was found a simple relationship of variables (glycolipids profile components) with the hormone T4 and TSH. But in terms of the statistically significant, it was found that only HDL level has a significance with gender, and only triglycerides have a significance with age. Isolated lipidomic profile components showed that 10 patients have elevated levels of all lipid profile components, while 42 patients showed elevated levels of hypercholesterolemia, Low-HDL, and High-LDL. These findings strongly indicate the prevalence of glycolipidemia in patients living in the Ma'an Governorate. In light of these results, it is necessary to create awareness programs focused on the pattern of nutrition, and physical activity, and to conduct genetic tests, because genetic problems are one of the factors causing high hyperlipidemia.

## Dodatak

### Authors' contributions

Prof. Omar Atrooz designed the study, wrote and reviewed the manuscript. Dr. Mazen Hiresh, Mohammad Atrooz, and Ghofran Hiresh, and Ihssan Atrooz coordinated the data collection, participated in the design of the manuscript, and revised the statistical analysis. Reham Alghonmeen and Aseel Alasoufi collected the data results of patients from the hospital and revised the manuscript. All authors read and approved the final version of the manuscript.

### Acknowledgments

We would like to thank all members of Ma’an Government Hospital for their help, support, and secured the ethical clearance to conduct the study. Also, we would like to thank Mr. Anees Hjazeen for performing the statistical analysis.

### Conflict of interest statement

All the authors declare that they have no conflict of interest in this work.
